# Role of peroral cholangioscopy and pancreatoscopy in the diagnosis and treatment of biliary and pancreatic disease: past, present, and future

**DOI:** 10.3389/fgstr.2023.1201045

**Published:** 2023-10-03

**Authors:** Harishankar Gopakumar, Neil R. Sharma

**Affiliations:** ^1^ Department of Gastroenterology and Hepatology, University of Illinois College of Medicine at Peoria, Peoria, IL, United States; ^2^ Parkview Cancer Institute, Advanced Interventional Endoscopy & Endoscopic Oncology (IOSE) Division, GI Oncology Tumor Site Team, Fort Wayne, IN, United States

**Keywords:** pancreatoscopy, IPMN - intraductal papillary mucinous neoplasm, cholangioscopy, SpyGlass direct visualization system, biliary stricture, cholangiocarcinoma

## Abstract

Peroral cholangiopancreatoscopy was described as early as the 1950s. However, the small caliber of these ducts and the technological limitations in developing slender, maneuverable, high-definition scopes posed a challenge. Peroral cholangiopancreatoscopy has now rapidly evolved. What began as dual-operator mother–daughter cholangioscopy systems that were fragile and difficult to use are now single-operator systems. The development of high-definition video cholangioscopes, along with improved flexibility and accessory technologies in recent years, has permitted single-operator, high-quality endoluminal examination and therapies of the biliary and pancreatic ducts. It is now an indispensable tool in the comprehensive diagnosis and definitive management of complex biliary and pancreatic conditions, such as indeterminate biliary strictures and difficult-to-remove biliary and pancreatic stones. With the enhanced imaging capabilities and refined maneuverability of the latest generation of cholangioscopes, the role of cholangiopancreatoscopy is expanding, with applications in advanced gall bladder drainage, accurate determination of tumor stage, cholangioscopy-directed tumor ablation, and selective biliary cannulation. In this review, we detail the evolution of this technology, the various approaches to peroral cholangiopancreatoscopy, and its established and emerging diagnostic and therapeutic indications. Furthermore, we discuss the current limitations and potential future applications of cholangioscopy and pancreatoscopy in managing various biliary and pancreatic pathologies.

## 1 Introduction

Cholangioscopy and pancreatoscopy allow direct endoscopic visualization of the biliary and pancreatic duct lumen. Direct access to the pancreatic and biliary ducts for cholangiopancreatoscopy can be obtained through an intra-operative, percutaneous, or endoscopic approach. These techniques thus permit diagnostic and therapeutic interventions in these difficult-to-access locations. Early attempts at these techniques beginning in the 1950s have been described in the literature by Roca et al. (1) Endoluminal examination of the bile duct was initially used in intra-operative evaluations to locate stones during common bile duct exploration (2). Intraoperative cholangioscopy can be performed during laparoscopic or open surgery. A cholangioscope can be inserted into the biliary or pancreatic duct, depending on the indication. For example, a trans-cystic or trans-ductal approach can be used for the cholangioscopic localization of stones during laparoscopic bile duct exploration for suspected choledocholithiasis (3). SpyGlass™ Discover (Boston Scientific, MA) is a novel cholangioscopy platform that provides minimally invasive, primarily laparoscopic access to the pancreaticobiliary system. This device, with its enhanced digital imaging capability, ability to provide a four-way deflection, dedicated irrigation channel, and an additional suction channel, is a significant improvement over standard surgical cholangioscopes (4).

Subsequently, with the advancement of image-guided transhepatic cholangiography, typically performed by an interventional radiologist, cholangioscopy has found a role as an adjunct technique during percutaneous transhepatic cholangiography for stricture and stone visualization and treatment. Percutaneous transhepatic cholangioscopy/pancreatoscopy is typically performed after a percutaneous transhepatic cholangiography for biliary drainage. Serial dilations of the percutaneous access point are then performed over several days, and the drainage catheter is left in place. Once the tract matures, the catheter is removed. A cholangioscope can then be inserted through a 12F sheath over a guidewire into the biliary tract under endoscopic and fluoroscopic visualization. The percutaneous approach is often the preferred approach in patients with an altered anatomy [owing to, for example, a Roux-en-Y hepaticojejunostomy or pancreaticoduodenectomy (Whipple procedure)] in whom it is often challenging to access the biliary tree endoscopically (5). Dedicated cholangioscopes for transhepatic cholangioscopy are available, such as the CHF-CB30L/S (Olympus Corporation). Spyglass Discover and peroral cholangioscopes such as Spyglass DS (Boston Scientific, MA) have also been used for transhepatic cholangioscopy/pancreatoscopy (6).

Currently, cholangioscopy and pancreatoscopy are most commonly performed via the peroral approach. Peroral cholangiopancreatoscopy (POCP) is performed by passing an endoscope through the mouth and gaining access to the biliopancreatic duct system across the ampulla of Vater. POCP is the preferred route when anatomic conditions are favorable. There are two broad approaches to performing a POCP: (i) a dedicated cholangioscope can be inserted through the working channel of a duodenoscope and passed across the ampulla of Vater to access the pancreatic or biliary duct system, or (ii) a direct peroral cholangiopancreatoscopy (DPOCP) can be performed, in which a single ultra-slim gastroscope is introduced perorally to the duodenum and then maneuvered across the ampulla of Vater into the biliary or pancreatic ductal system. The former method was initially introduced as a dual-operator system, with one endoscopist controlling the duodenoscope while a second operator maneuvered the cholangioscope. These systems were referred to as “mother–daughter” peroral cholangioscopes and were a significant advancement. However, they were cumbersome and fragile, limiting widespread adoption. Subsequently, the more efficient single-operator systems where the duodenoscope and the cholangioscope are controlled by the same operator were introduced. With further technological improvement and the addition of high-definition digital imaging, both single-operator peroral cholangiopancreatoscopy and DPOCP have greatly improved our diagnostic and therapeutic capabilities for managing complex biliopancreatic pathologies. **Table 1** summarizes the various approaches to performing cholangiopancreatoscopy and examples of the multiple available devices.

**Table 1:** Approaches to performing cholangiopancreatoscopy and examples of the various devices that are currently available in the market.

Approach to cholangiopancreatoscopy.		Examples of commercially available devices	Comments
Per-Oral Cholangioscopes	Single Operator Cholangioscopy	Spyglass Legacy (Boston Scientific) Spyglass DS (Boston Scientific) Spyglass DS II (Boston Scientific)	Single use disposable cholangioscopes
	Dual Operator Cholangioscopy	CHF-BP30 (Olympus) FCP- 9P (Pentax)	Reusable cholangioscopes that can be inserted through the working channel of a standard duodenoscope
	Direct Peroral Cholangioscopy	GIF-XP190N (Olympus) GIF-XP 180N (Olympus) GIF-180N (Olympus) PEF-V transnasal (Olympus) EG-530N (Fujinon) EG-530NP tansnasal (Fujinon) EG 1690K (Pentax) CHF-YOO10, Prototype Multibending (MB) ultra slim endoscope (Olympus).	Reusable ultra-slim gastroscopes
Percutaneous Transhepatic Cholangioscopes		CHF-CB30L/S (Olympus) Spyglass Discover (Boston Scientific) Spyglass DS (Boston Scientific) Spyglass DS II (Boston Scientific)	Single operator per-oral cholangioscope system Spyglass (Boston Scientific) can also be used for percutaneous transhepatic cholangioscopy.
Intra-Operative Cholangioscopes		Spyglass Discover (Boston Scientific) FCN- 15X (Pentax)	

### 1.1 Per-oral Cholangiopancreatoscopy platforms

#### 1.1.1 Dual operator POCP

A system for peroral cholangioscopy was developed in the 1970s, consisting of a large-channel duodenoscope and a second smaller cholangiopancreatoscope that could be passed through the working channel of the duodenoscope (7–9). These were called “mother–baby” or “mother–daughter” systems and required two endoscopists to maneuver, one controlling the duodenoscope and the other operating the cholangioscope. The original dual-operator systems were fiber optic and prone to frequent damage (10). Although the outcomes with this system were encouraging, it was not widely adopted as it was cumbersome, expensive, and labor-intensive. The fiber-optic peroral cholangioscopes CHF-BP30 (Olympus Corporation, Central Valley, PA) and FCP-9P (Pentax Corporation, Montvale, NJ) are currently available in the USA and can be used for dual-operator POCP (2).

#### 1.1.2 Single operator POCP

**Figure 1:**
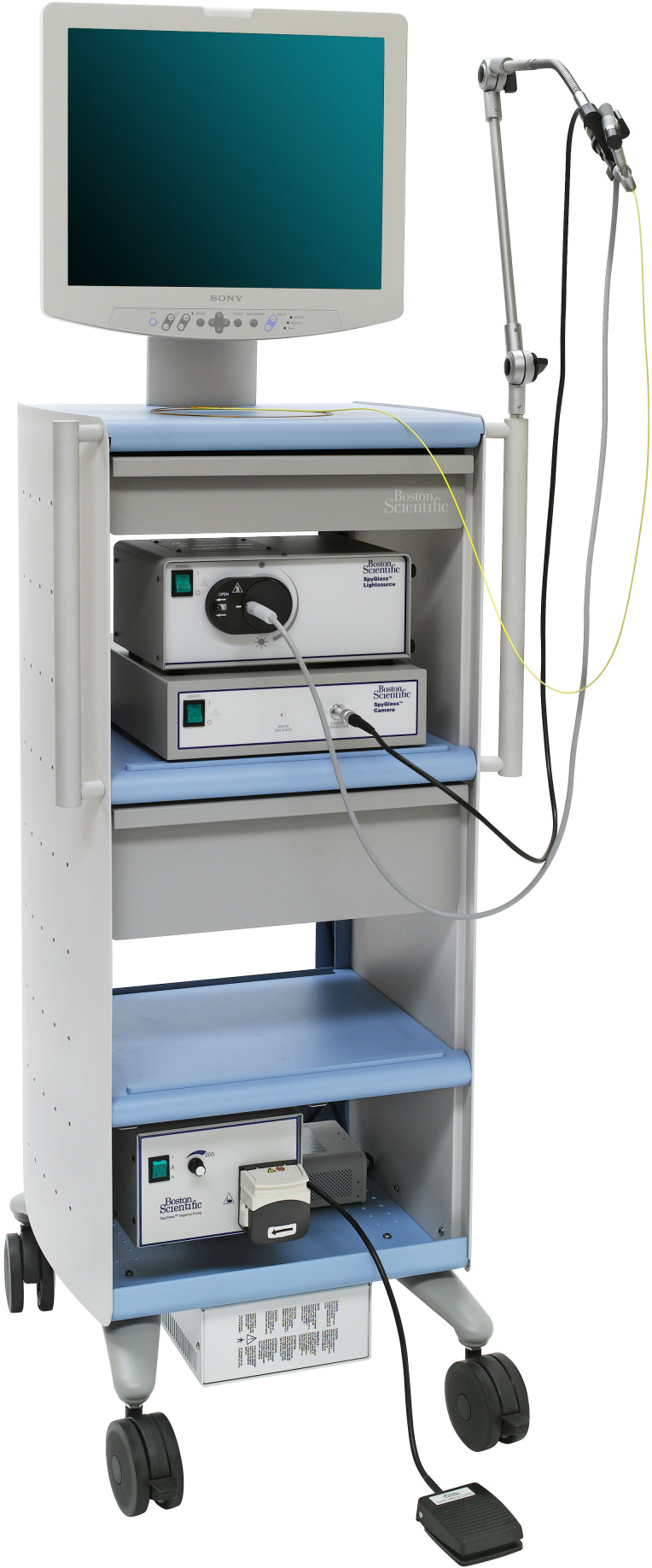
The SpyGlass Legacy system.

**Figure 2:**
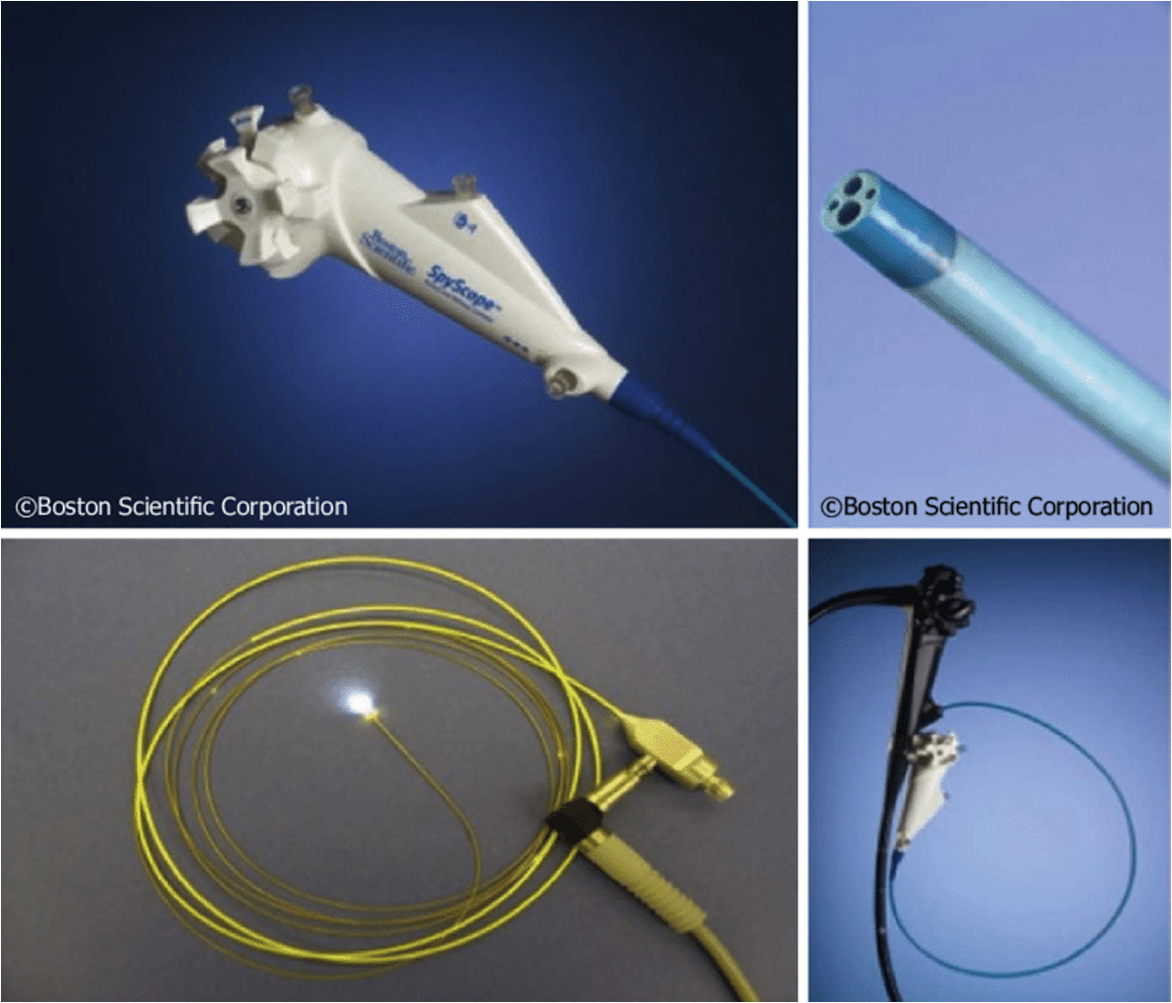
Components of the SpyGlass Legacy system.

**Figure 3:**
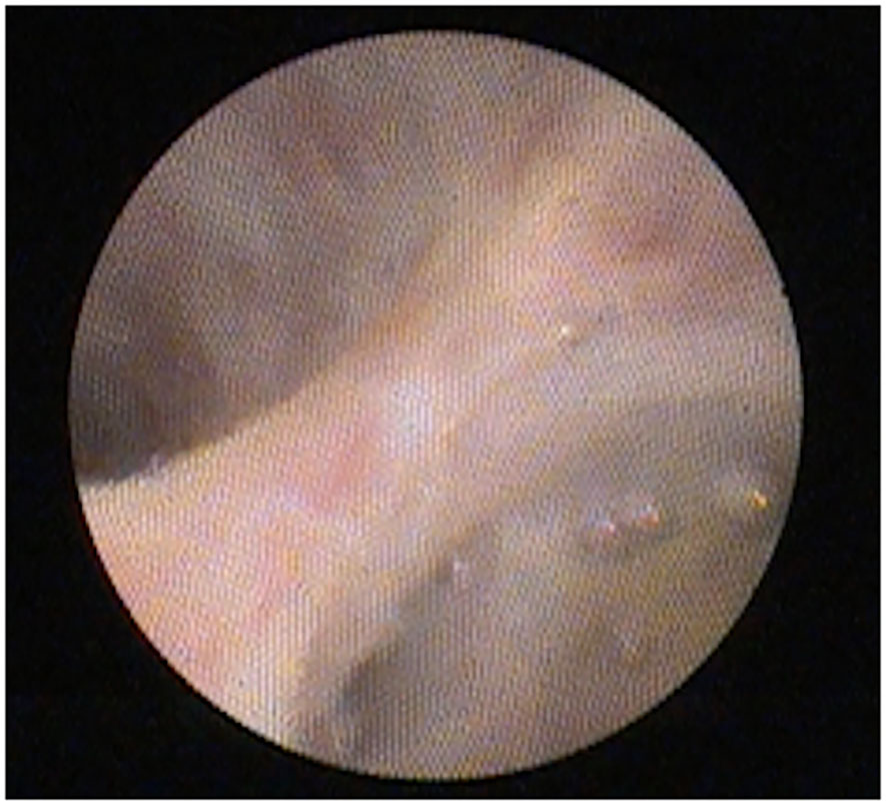
Quality of image obtained using the SpyGlass Legacy system.

**Figure 4:**
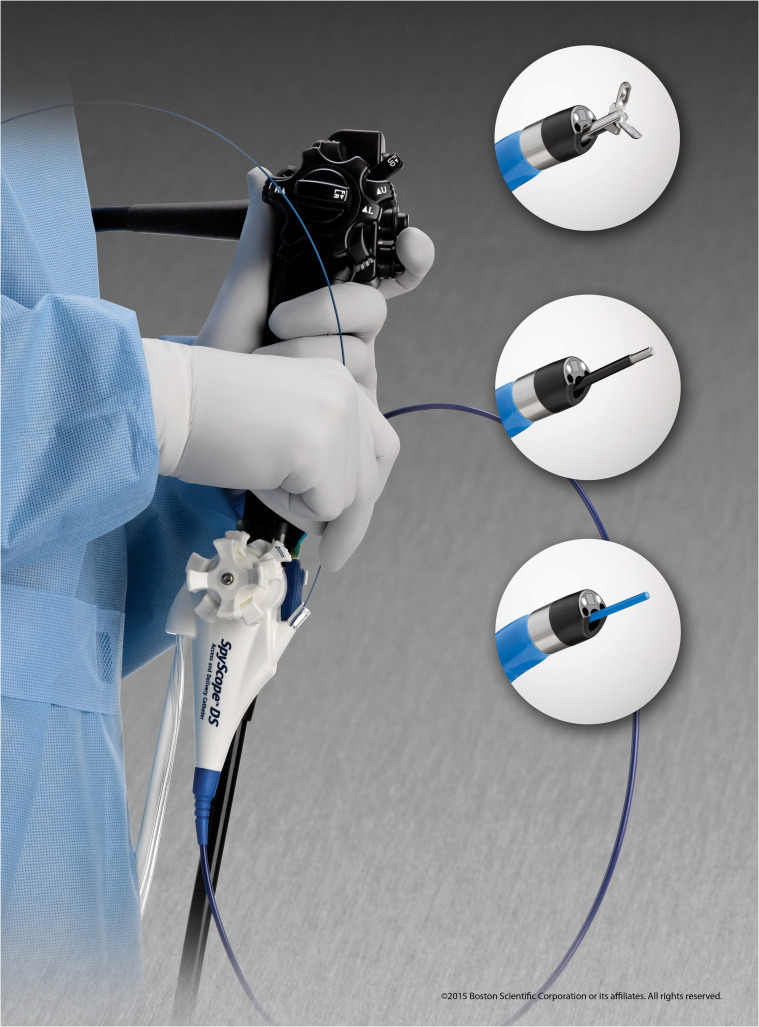
Spyglass DS II with the SpyScope DS II delivery and access catheter.

In 2007, a single-operator cholangioscope (SOC) system, SpyGlass® (Boston Scientific, Marlborough, Massachusetts, USA), was introduced (**Figure 1**). This included a reusable fiber-optic probe that is passed through a disposable catheter and can then be introduced through the working channel of a therapeutic duodenoscope and advanced over a catheter across the papilla. The components of this system are shown in **Figure 2**. This SOC system also had the advantage of four-way tip deflection, allowing better maneuverability. This was called the SpyGlass Legacy^®^ (Boston Scientific, Marlborough, Massachusetts, USA) system. Although a significant improvement compared with the DOC systems, it still suffered from suboptimal image quality (**Figure 3**), a prolonged setup, and limitations in probe durability. There was also a reported lack of coordination between the directional control dial movements and optical fiber visualization (11). With technological advancements, a newer, fully digital single-operator cholangioscope system called the SpyGlass DS® system with a SpyScope DS catheter was released in 2015. This substantially improved image quality, maneuverability, and the ability to accept a broader range of accessories than its predecessor (**Figure 4**) (12). In 2019, the latest iteration of the Spyglass system, called the Spyglass DS II with SpyScope DS II delivery and access catheter, was launched, which increased the resolution by 2.5 times. In addition, the footprint of the processor and system is much smaller, allowing for easier incorporation into the endoscopy suite.

#### 1.1.3 Direct POCP

In direct peroral cholangioscopy or pancreatoscopy, a narrow-caliber forward-viewing endoscope is inserted through the mouth, passed into the duodenum, and then maneuvered across the ampulla of Vater to access the biliary or pancreatic duct system. This technique requires a prior large sphincterotomy with or without balloon dilation. It was first described in 1977 by Urakami et al. (13) However, its adoption remained limited owing to the difficulty in traversing the sphincter of Oddi to gain access to the biliary or pancreatic duct using an ultra-slim floppy gastroscope and the poor image quality of earlier fiberoptic scopes (14). With the introduction of high-definition ultra-slim upper endoscopes with narrow-band imaging capability, direct peroral cholangioscopy has gained popularity (15). The early advantages of D-POCP were the greater availability and lower operating cost of the reusable upper endoscopes compared with the more expensive single-use high-definition video cholangioscopes (14). Other advantages of D-POCP include better image quality, the larger size of the working channel (2.2 mm compared with 1.2 mm in dedicated cholangioscopes), and the ability to perform irrigation, suction, and therapeutic maneuvers simultaneously (14). New devices are in development to preserve the advantages offered by DPOCP while eliminating some of the challenges to its use. Lee et al. compared a new multi-bending (MB) ultra-slim endoscope to the conventional ultra-slim endoscope for cholangioscopy (16). The MB ultra-slim scope was technically successful in 89.1% of patients, which was significantly higher than the success rate (30.4%) in the conventional group. The procedure time of direct POC using free-hand biliary insertion of the endoscope was significantly shorter in the MB group than in the conventional group (16).

## 2 Diagnostic and Therapeutic Applications

The infusion of contrast media into the ampulla of Vater during surgery to visualize the biliary and pancreatic duct system was first reported in the 1950s. In 1965, Rabinov et al. described the first non-operative injection of contrast into the ampulla of Vater using a peroral tube under fluoroscopic guidance (17). In 1968, McCune et al. described one of the first attempts at injecting contrast into the ampulla of Vater under direct endoscopic visualization (18). Endoscopic retrograde cholangiopancreatography (ERCP) has improved greatly since the 1960s and is now the primary diagnostic and therapeutic method for most biliary and pancreatic pathologies. However, ERCP is still an indirect method of evaluating the biliary or pancreatic ductal anatomy as it does not offer direct visualization of the mucosa or the lumen. Cholangiopancreatoscopy provides the additional benefit of direct visualization and the ability to perform novel diagnostic and therapeutic interventions.

### 2.1 Applications of Cholangioscopy

#### 2.1.1 Difficult biliary duct stones

**Figure 5:**
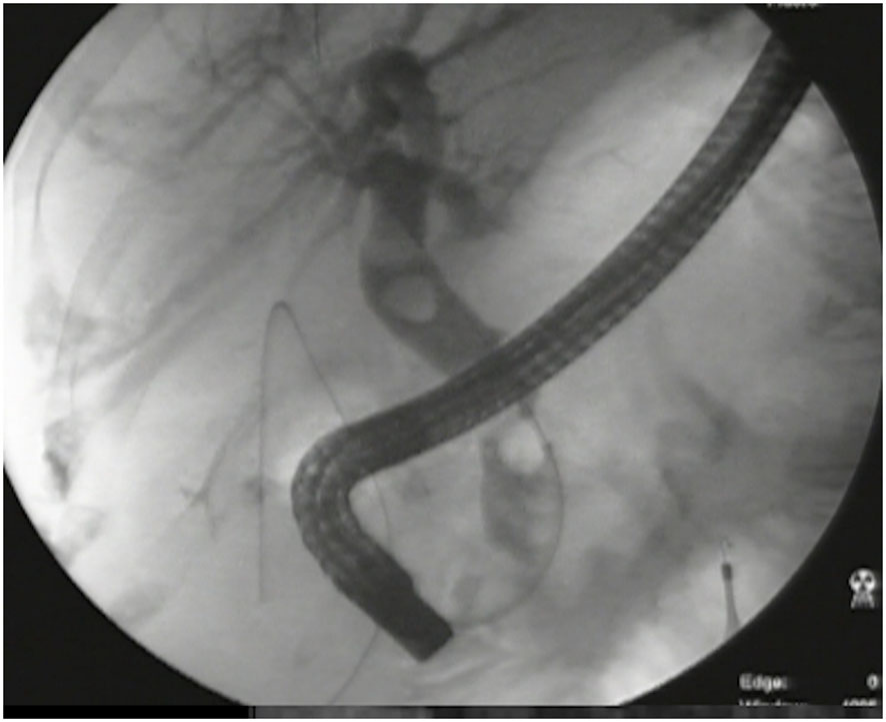
Large stones in the CBD seen on a cholangiogram.

Choledocholithiasis is the presence of stones in the biliary ductal system. About 15% of individuals with symptomatic cholelithiasis have choledocholithiasis. It can be asymptomatic in a small proportion of patients but may result in obstructive jaundice, ascending cholangitis, pancreatitis, or cystic stump leaks following cholecystectomy (19). ERCP with a sphincterotomy and stone extraction is universally the most common procedure for managing choledocholithiasis. However, the stones can be challenging to extract in a small subgroup of cases, requiring additional measures. **Figure 5** shows large stones in the CBD as seen on a cholangiogram. Characteristics that make stones difficult to remove with standard ERCP include stone size (≥ 15 mm in size), impaction, a high number of stones, intrahepatic location, stone shape (i.e., piston shaped), a hard consistency, the presence of bile duct stricture distal to the stone, and unusual biliary anatomy (a sigmoid-shaped common bile duct) (20). These difficult stones can be classified as extrahepatic and intrahepatic stones, based on location. Extrahepatic stones are located distal to the confluence of the right and left hepatic ducts. In contrast, intrahepatic stones are located proximal to the confluence, and the condition is referred to as hepatolithiasis. Laparoscopic common bile duct exploration (LCBDE) with cholecystectomy has also been proposed as a treatment option if surgical expertise is available (19). However, with advances in tools for ERCP and the advent of high-definition cholangioscopy, the need for LCBDE has declined to almost zero in high-volume and tertiary centers.

**Figure 6:**
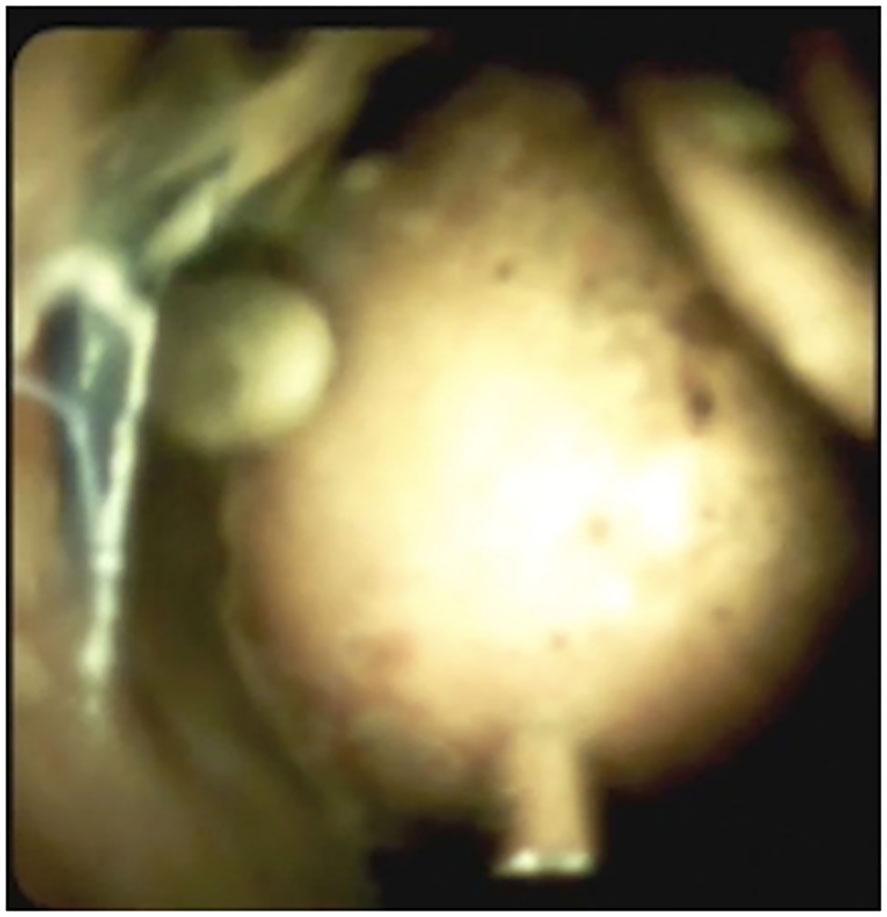
Cholangioscopic appearance of a large CBD stone before EHL.

**Figure 7:**
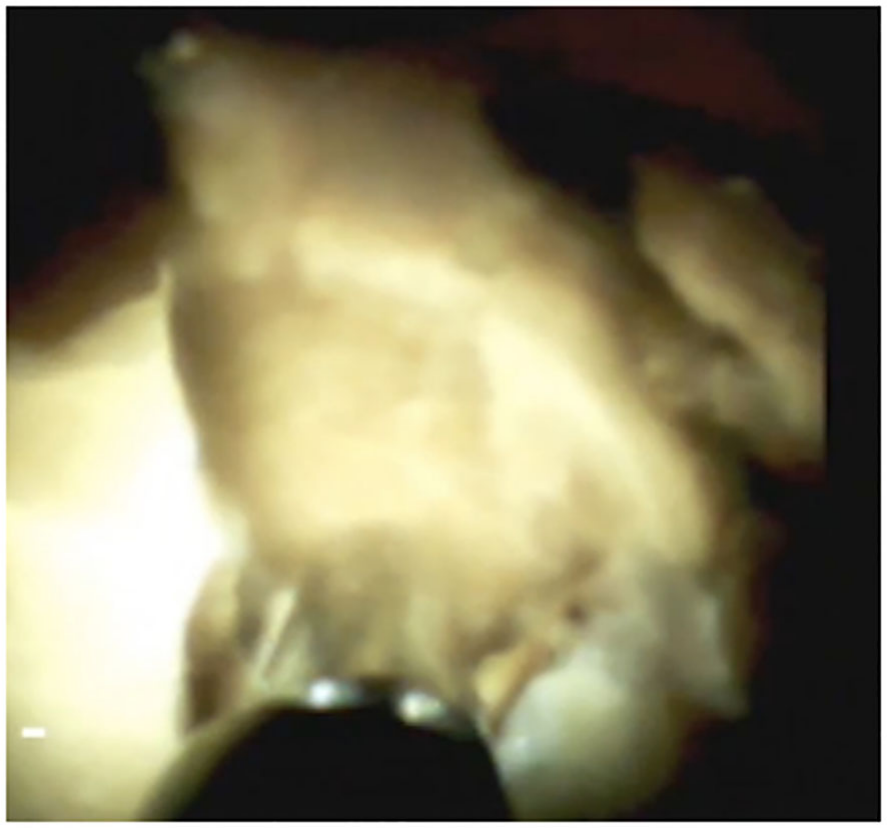
Cholangioscopic view of a fragmented CBD stone after EHL.

**Figure 8:**
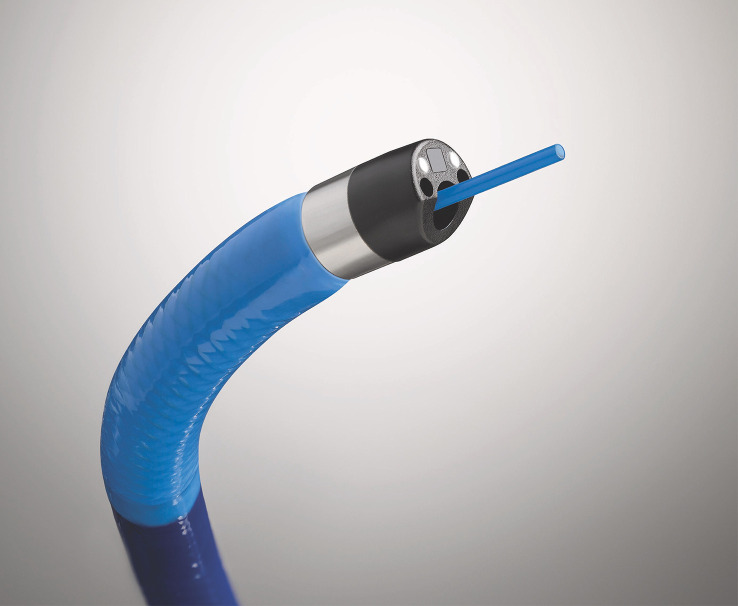
SpyScope with the laser lithotripsy probe tip.

Peroral cholangioscopy with intraductal lithotripsy is a very effective treatment option, with extrahepatic stone clearance rates of 71%–100% reported in the literature (20). Electrohydraulic lithotripsy (EHL) and laser lithotripsy (LL) are the techniques used to fragment large intraductal stones. **Figure 6** shows the cholangioscopic appearance of a large CBD stone before EHL and **Figure 7** shows stone fragmentation following EHL. SpyScope with the laser lithotripsy probe tip is shown in **Figure 8**. Peroral cholangioscopy for intraductal lithotripsy is usually performed using the standard peroral approach, accessing the CBD across the ampulla of Vater. However, in some cases, such as those with a surgically altered anatomy or duodenal obstruction, transpapillary access to the CBD may not be achievable and endoscopic ultrasound (EUS)-guided biliary access methods, such as hepaticogastrostomy, hepaticojejunostomy, and choledochoduodenostomy, have to be employed. Peroral transluminal cholangioscopy (PTLC) is an advanced novel technique in which cholangioscopy is performed by accessing the bile duct through the fistula between the bile duct and visceral lumen. In a study of 42 consecutive patients with difficult bile duct stones who underwent cholangioscopic EHL, eight of whom were PTLC, the rate of complete stone clearance was 98% (21). In a study of 94 patients who underwent POC-guided lithotripsy (both EHL and LL), Alexandrino et al. reported complete ductal clearance in 93 of 94 patients (98.94%) (22). A previous systematic review of 33 studies on difficult bile duct stones reported an overall estimated stone clearance rate of 88% (23). The estimated rate of severe adverse events in this meta-analysis was low at 1%, with an overall adverse events rate of 7%. The evidence so far on using POC-guided lithotripsy to manage difficult bile duct stones has been very encouraging.

Hepatolithiasis is the presence of calculi in the intrahepatic bile ducts. Its pathogenesis is not fully understood and it is more common in East Asia, where studies have reported up to 53.50% prevalence in patients with cholelithiasis (24). Percutaneous transhepatic cholangioscopic lithotomy (PTCSL) and hepatic resection are the two main treatment options for this challenging and often debilitating condition. Peroral cholangioscopic lithotomy (POCSL) can be a less invasive treatment option for intrahepatic stones. However, it is sometimes limited by the inability of currently available cholangioscopes to pass through the narrow intrahepatic bile ducts and strictures. Okugawa et al., in a case series on 36 consecutive patients who underwent POCSL to treat hepatolithiasis, reported a complete stone removal rate of 64%. This study used a mother–daughter POC and included patients with stones located only in the smaller peripheral IHBDs (25). Mansilla-Vivar et al. reported a clinical success rate of 57% in a small cohort of seven patients using a single-operator cholangioscope system (26). Complete stone clearance rates have been higher with PTCSLs than with the cholangioscopic approach, with rates of 88.40% reported by Cheng et al. in a large case series involving 190 patients (27). In an evaluation of long-term results and recurrence factors after operative and non-operative treatments for hepatolithiasis, Cheon et al. reported complete stone clearance rates of 83.3% with hepatectomy, 63.9% with PTCSL, and 57% with POCSL. This study also observed higher recurrence rates and cholangitis with non-surgical management of hepatolithiasis (28). Thus, current evidence suggests that POC-lithotripsy can be an effective treatment strategy for hepatolithiasis, but careful patient selection is imperative.

#### 2.1.2 Ductal Clearance

**Figure 9:**
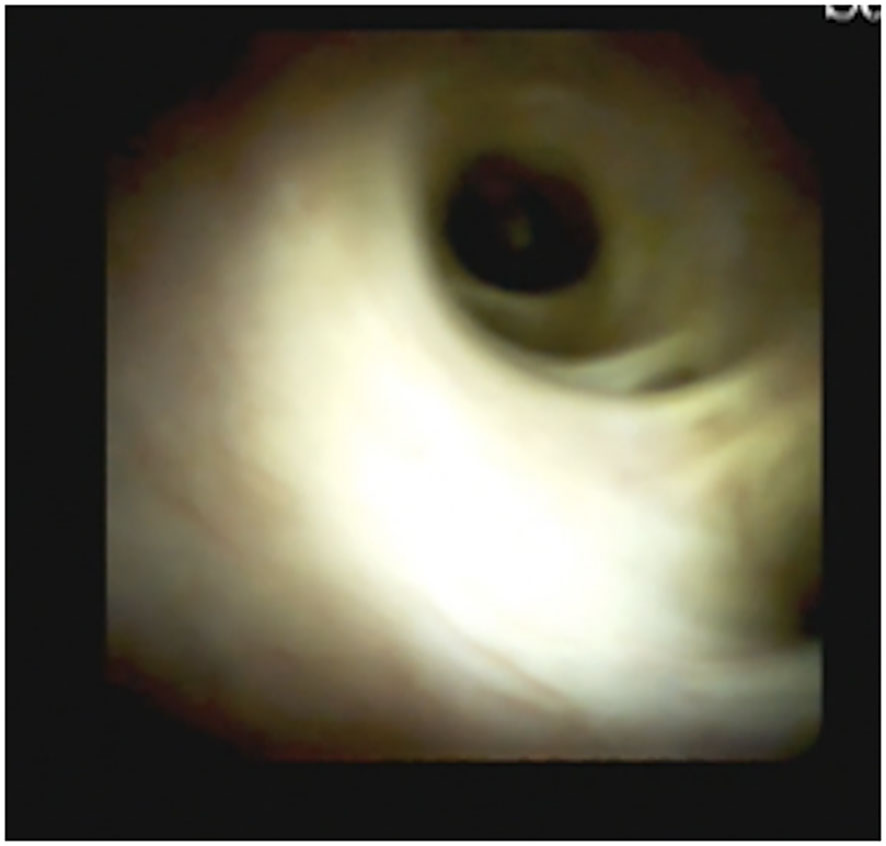
Cholangioscopy showing complete ductal clearance.

Evaluation of ductal clearance after endoscopic treatment of choledocholithiasis is usually performed using an occlusion cholangiogram after stone retrieval. However, cholangiography is imperfect and small stones can be overlooked. Multiple studies have reported rates of residual common bile duct stone of over 20% (29–32). In a study of 36 patients who underwent POC after ERCP with negative occlusion cholangiogram, seven (22.50%) had residual CBD stones (30). Yang et al. detected residual stones in 19 of 79 (25.30%) patients using DPOC after a negative cholangiogram. Out of these 19 patients, 13 were noted to have multiple stones. All the stones were less than 5 mm in size and could be removed at the time of cholangioscopy (31). Eleven of 34 (32.40%) patients with no filling defects found on an occluded balloon cholangiogram who underwent single operator cholangioscopy were found to have residual stones, and these could be successfully removed in 10 cases true impact on the long term clinical outcome of small < 5 mm retained stones is unclear, and whether they need to be consistently evaluated is still a matter of debate. Itoi et al. demonstrated that large bile duct stones, juxta papillary diverticulum, and the use of mechanical or electrohydraulic lithotripsy were significantly associated with residual stones (33). Overall, this shows that POC is a valuable tool for assessing ductal clearance and treating residual stones after conventional ERCP-guided stone extraction (**Figure 9**). Patient selection for this indication should be individualized based on risk factors to justify the added cost and procedural risk.

#### 2.1.3 Indeterminate biliary strictures

**Figure 10:**
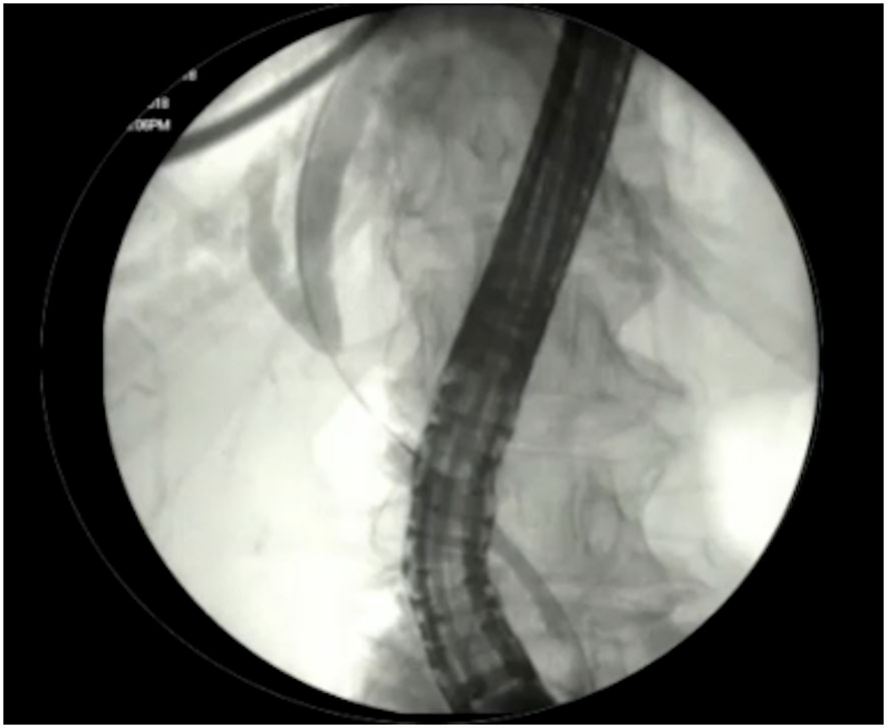
Biliary stricture seen on ERCP.

Biliary strictures can be caused by pathologies both intrinsic and extrinsic to the bile duct (**Figure 10**) (20). Indeterminate biliary strictures (IDBS) are defined as strictures without a definite diagnosis after cross-sectional imaging with/without non-diagnostic brush cytology results on ERCP (34, 35). Given the high likelihood of malignancy, especially in individuals presenting with jaundice and the grave prognosis of advanced disease, early surgical management is critical for a favorable outcome (35, 36). However, up to 20% to 30% of patients undergoing surgical resection for suspected malignant biliary strictures can have a benign etiology (35, 36). Biliary surgeries can have significant morbidity, while benign strictures can be treated with serial endoscopic dilations, leading to favorable outcomes. Therefore, an accurate diagnosis can be critical to avoid unwarranted surgeries. Traditionally, ERCP with tissue acquisition (brush cytology with or without trans-papillary biopsy) has been the primary means of evaluating biliary strictures. This has excellent specificity (99%) but suffers significantly from low sensitivity (45% for brush cytology and 48% with trans-papillary biopsy), resulting in non-diagnostic studies (37, 38). One of the major limitations of ERCP-guided biliary duct biopsy is that, although it is performed under fluoroscopic guidance, it is essentially a blind process owing to the lack of direct mucosal visualization (39). The two major malignancies that can present with biliary strictures are cholangiocarcinoma and extrinsic compression or direct invasion from pancreatic adenocarcinoma (36, 40). Other less common malignant etiologies that can present with biliary strictures include gall bladder cancer obstructing the bile duct, ampullary cancer growing into the bile duct, malignant periportal lymph nodes, and metastatic cancers of the pancreas or liver (40). Benign etiologies for biliary strictures include iatrogenic bile duct injuries (following liver transplant or biliary surgeries), chronic pancreatitis, Mirizzi syndrome, ischemic cholangiopathies, primary sclerosing cholangitis (PSC), autoimmune conditions (autoimmune pancreatitis and autoimmune cholangitis), infectious diseases (tuberculosis and parasitic infections), radiation-induced biliary duct sclerosis, and, rarely, mild structuring from long-term retention of choledocholithiasis (41).

Pancreatic adenocarcinoma is a major consideration when patients present with jaundice and distal common bile duct obstruction, while cholangiocarcinoma is the primary concern when patients present with strictures in the mid and proximal extrahepatic bile ducts. Although pancreatic cancer may be seen as a mass-like lesion on cross sectional imaging, studies have reported a miss rate of up to 50% on computed tomography (CT) and magnetic resonance imaging (MRI) (40). EUS can be used for diagnosis in such situations to identify a mass-like lesion, but obtaining a cytological or histologic diagnosis with EUS-FNA or EUS-FNB is operator dependent and can be challenging in less experienced hands. Various laboratory tests, such as serum CA 19–9 and carcinoembryonic antigen (CEA), lack specificity, as do other tumor markers such as transthyretin (TTR), interleukin-6 (IL-6), and matrix metalloproteinase-7 (MMP-7) (40).

POC helps to evaluate indeterminate biliary strictures by allowing the direct visualization of the biliary tract, including a detailed visual assessment of the mucosa, and also by permitting targeted biopsies (34).

#### 2.1.4 Direct visualization

**Figure 11:**
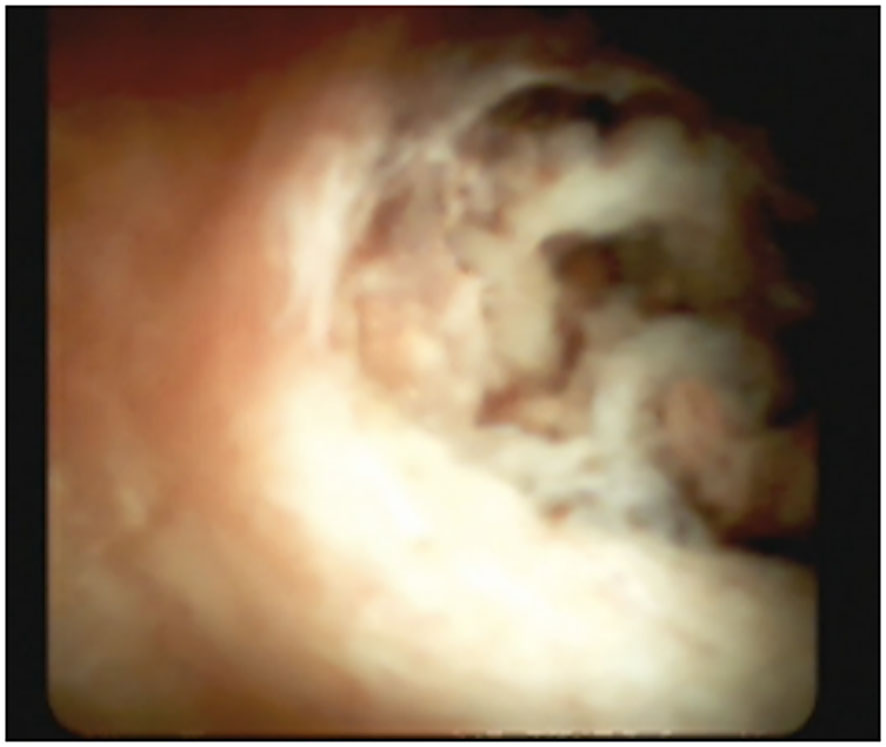
Appearance of cholangiocarcinoma on cholangioscopy.

With the availability of high-definition wide-angle views using newer digital single-operator cholangioscopes, considerable advances have been made in the diagnostic accuracy of visual impressions. Visual impression has been shown to have better sensitivity than biopsy but lower specificity (38). Endoscopic features suggestive of malignancy include irregularly dilated and tortuous vessels (tumor vessels), papillary or villous mucosal projections, and intraductal nodules or masses (**Figure 11**) (2, 42). In a systematic review and meta-analysis of the efficacy of SOC in the visual interpretation of indeterminate biliary strictures, de Oliveira et al. reported an impressive pooled overall sensitivity and specificity of 94% (95% CI 89%–97%) and 95% (95% CI 90%–98%), respectively, with a positive and negative likelihood ratio of 15.02 (95% CI 5.21–44.33) and 0.08 (95% CI 0.04–0.14), respectively, from 283 total procedures (12). A systematic review that involved all types of cholangioscopes had shown a sensitivity and specificity of 67%–100% and 49%–100%, respectively, for the use of visual impressions in diagnosing malignancy in indeterminate biliary strictures (43). Narrow-band imaging (NBI) with magnifying endoscopy has significantly improved the ability of clinicians to delineate surface structures. In one of the earliest studies on NBI in cholangioscopy, Itoi et al. demonstrated the superior ability of NBI to demarcate lesion margins and blood vessels compared with conventional cholangioscopy with white-light imaging (WLI) (44). Tumor vessels (irregularly dilated and tortuous vessels) have the highest specificity for malignant lesions, with studies reporting a specificity of 100% (45). In earlier studies, sensitivity values were low owing to the lower quality of images that limited the ability to identify these subtle findings. For example, Kim et al., in their study from 2000, could observe this pathognomonic finding of tumor vessels in only 25 of 41 patients with biliary malignancy (sensitivity 61%), although all patients with this observation had malignancy (specificity 100%) (45). With the improvement in imaging technologies, this rate has overall improved significantly. In a recent study from 2022, in 71 patients who underwent DPOC with NBI for evaluation of indeterminate biliary strictures, WLI correctly identified 24 of 32 malignant lesions whereas NBI correctly identified 28 of 32 malignant lesions (sensitivity and specificity of 75% and 82.90%, respectively for WLI and of 87.5% and 91.40% for NBI, respectively). Cholangioscopy, with the addition of NBI, can thus be an excellent tool for evaluating indeterminate biliary strictures.

#### 2.1.5 Cholangioscopy-directed targeted biopsies

**Figure 12:**
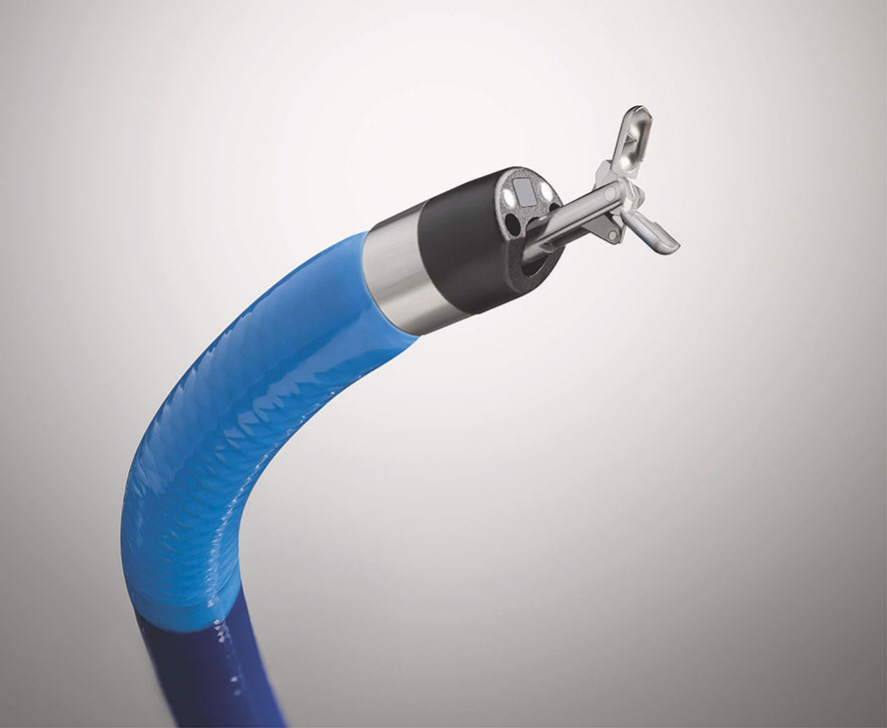
SpyBite forceps® (Boston Scientific).

**Figure 13:**
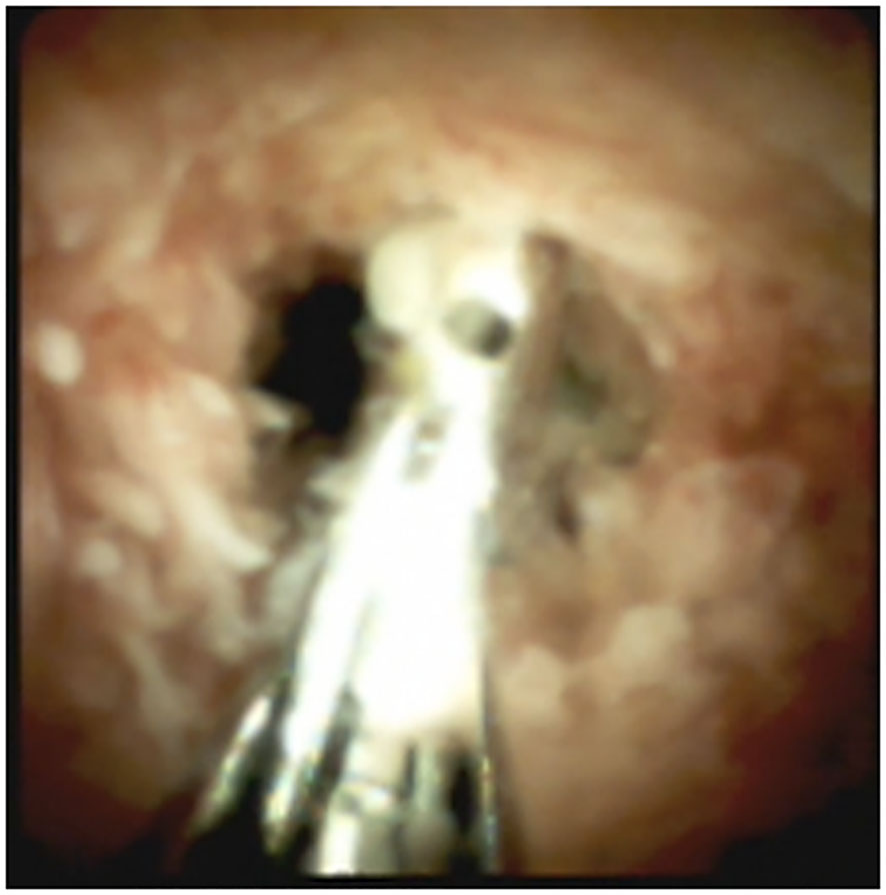
Cholangioscopic view of a luminal biopsy from the CBD using the SpyBite forceps.

Although visual impressions have excellent sensitivity, their specificity is suboptimal, and tissue diagnosis remains the gold standard. Before the introduction of SOC systems, cholangioscopy-directed biopsies were challenging, and their use was limited. The introduction of SOC, along with the use of forceps (**Figure 12**—SpyBite forceps® (Boston Scientific, Marlborough, Massachusetts, USA)), improved the diagnostic yield and accuracy of cholangioscopy-directed biopsies. **Figure 13** shows the cholangioscopic view of a luminal biopsy from the CBD using SpyBite forceps. Newer SpyGlass DS digital cholangioscopes have further improved diagnostic yield and accuracy. In a large multicenter study, Navaneethan et al. reported a sensitivity of 85% and specificity of 100% using the SpyGlass DS SOC system for malignant lesions of the biliary tract (11). This was an improvement compared with the pooled sensitivity of 84.5% and specificity of 82.6% reported in a systematic review of the first-generation fiberoptic SOC system for evaluating malignancy in biliary strictures. This study also reported that an adequate histologic sample was obtained for 97% of patients. A recent systematic review by Kulpatcharapong et al. on the performance of different cholangioscopes for malignant biliary strictures showed improved sensitivity of cholangioscope-directed biopsy with digital cholangioscopes (both SOC and DPOC) compared with previous fiber-optic scopes (43). Results from a large prospective multinational registry that included patients from Asia, the Middle East, and Africa showed that adequate diagnostic samples could be obtained in 169/182 (92.90%) of cholangioscopy-directed forceps biopsies (46). SpyBite forceps® (Boston Scientific, Marlborough, Massachusetts, USA) are ultra-fine endoscopic biopsy forceps that can typically obtain only a small amount of tissue. This is one of the reasons for the lower sensitivity and diagnostic yield of POC-guided biopsies. An improved biopsy device was recently released for the SpyGlass DS system (SpyBite MAX; Boston Scientific). This devices features improvements including a 10% increase in cup volume, micro teeth at the tip of the forceps to improve issue gripping, and side holes to avoid contamination by liquid components such as blood and bile (39). A prospective study comparing SpyBite MAX to SpyBite forceps found that tissue sample size was larger in the SpyBite max group. It also required fewer biopsies to obtain a visible core sample. However, this did not result in a significant difference in diagnostic accuracy (39). Biopsy using POC-guided forceps is the ideal approach to evaluate an indeterminate biliary stricture, particularly for malignancy, given its very high specificity. Advancements in dedicated biopsy forceps can improve tissue yield and enhance sensitivity.

#### 2.1.6 Cholangiocarcinoma

##### Diagnosis and staging

Cholangiocarcinoma (CCA) is rare, but it is the most common biliary tract cancer arising from any part of the biliary tree, with an incidence of 0.3–6.0 cases per 100,000 persons (47). Based on the location, it is classified as intrahepatic (10%–20% of cases), perihilar (50%–60% of cases), or distal (20%–30% of cases) (48). Surgical management is the only curative option, but its efficacy is limited to early-stage disease (47). ERCP-directed biliary brushings have been the primary diagnostic modality employed to obtain tissue diagnosis for perihilar and distal extrahepatic cholangiocarcinoma. This technique has significant limitations, including desmoplasia, which results in only a few cells being collected, causing sampling errors and unacceptable false negative results. The sensitivity of conventional cytology in perihilar cholangiocarcinoma is approximately 20% (49). Percutaneous or EUS-guided biopsies of primary lesions in patients who are potential candidates for treatment with curative intent are discouraged due to the potential for tumor spread or seeding (49). Cholangioscopy, owing to its ability to directly visualize the biliary lumen, can be used to diagnose and define the extent of biliary involvement in cholangiocarcinoma (CCA). This is particularly important in Klatskin’s tumors, which involve the hepatic hilum (left and right main hepatic ducts). On cholangioscopy, cholangiocarcinoma presents as “frond-like” or “finger-like” projections and hypervascularity of the biliary mucosa. Cholangiocarcinoma can be identified in some cases of “adenocarcinoma of unknown primary.” The American Joint Commission on Cancer (AJCC) Cancer Staging Manual’s 7th and 8th editions define non-resectable cholangiocarcinoma as a lesion that involves the secondary radicals in the left and right system bilaterally (50). This can often be accurately defined by cholangioscopy, allowing for accurate peri-operative staging. Cholangioscopy can also identify “skip lesions” and alter surgical management when used pre-operatively. In a study by Tyberg et al., 105 patients underwent cholangioscopy for pre-surgical mapping. The findings from cholangioscopy resulted in less extensive surgery in six patients and the avoidance of surgery in 25 patients. Of these 25, 12 patients were found to have a more extensive disease, thus making them unsuitable for surgical intervention (51). Given the significant morbidity associated with pancreaticobiliary surgery, information from cholangioscopy can assist in accurate surgical planning to maximize the chance of attaining R0 resection and avoiding surgery in those who might not benefit from it.

##### Therapeutic application

POC-directed radiofrequency ablation (RFA) and POC-directed photodynamic therapy (PDT) have been studied as therapeutic options for locoregional therapy to manage unresectable extrahepatic CCA. Biliary obstruction is one of the main therapeutic challenges in managing perihilar and extrahepatic cholangiocarcinoma. Ensuring biliary patency and adequate drainage is the primary goal of endoscopic therapy for CCA. The goal of biliary drainage should be from > 50% liver volume as this has been shown to increase survival (119 days vs. 59 days, *p*=0.005) (52). The 2021 American Society of Gastrointestinal Endoscopy (ASGE) guidelines recommend that for patients with unresectable malignant hilar cholangiocarcinoma, the ultimate decision regarding the palliative draining strategy should be based on the patient’s preferences, disease characteristics, and local expertise (53). Stent occlusion due to tissue in-growth, epithelial hyperplasia, biofilm deposition, biliary sludge, and granulation tissue formation is a common complication encountered during the course of endoscopic management of biliary strictures (54). POC-directed RFA and PDT have been shown to reduce the incidence of stent occlusion and prolong stent patency.

For intraductal RFA, an electrode is introduced into the target lesion and a high-frequency alternating current is applied to induce ionic agitation in cancer cells. This results in coagulation necrosis and cellular death at the tumor site once the target temperature exceeds 48°C–50°C (55). This also releases intracellular components, including heat shock proteins that activate antigen-presenting cells and ultimately enhance local immunity against the tumor (54). The two available devices for intrabiliary RFA are the Habib HPB-RF probe® (Boston Scientific Corp., Marlborough, MA, USA) and the ELRA RF catheter® (Taewoong Medical, Gyeonggi-Do, South Korea) (54). In a comprehensive review of endoscopic RFA for unresectable malignant biliary strictures involving 12 studies and 318 patients, Larghi et al. reported only a few cases of failures (54). A systematic review and meta-analysis by Sofi et al. comparing a combination of RFA and biliary stenting with biliary stenting alone for malignant biliary strictures reported significantly longer stent patency in patients with cholangiocarcinoma treated with RFA, with a pooled weighted mean difference of 42.7 days (95% CI 17.19–68.1 days). The only adverse event more frequently encountered in the RFA group was abdominal pain (31% in the FRA group vs. 20% in the control group) (56). Many of these studies included patients treated with ERCP-guided and PTC-guided RFA. POC-directed RFA to treat complex non-operable cholangiocarcinoma, resulting in the maintenance of biliary drainage and stent patency, has been reported (57).

Photodynamic therapy (PDT) involves the intravenous administration of a photosensitizer that preferentially accumulates in neoplastic tissue. This is typically administered approximately 24 hours before the POC procedure. Activation of the photosensitizer is achieved by endoscopically applying light energy, typically a laser, that causes a photochemical reaction resulting in ischemia and necrosis of the neoplastic cells (58). A systematic review by Chen et al. compared the use of PDT alongside stenting with stenting alone. It showed the 1-year survival rate of the PDT-with-stent group to be significantly better (56%) than that of the control group (25%) (58). Most of the studies evaluating PDT have been on ERCP-directed PDT; however, it has been proposed that a cholangioscopy-guided placement of the laser probe can result in better accuracy and hence improved outcomes (59). In a retrospective study by Talreja et al. comparing PDT with and without cholangioscopic guidance, the median survival rate for the PDT-only group was 200 days and the median survival rate for the PDT-with-SOC group was 386 days, although this difference did not reach statistical significance (*p* = 0.45) (59).

Most data on RFA and PDT for biliopancreatic malignancies are from ERCP-guided treatment. However, findings from recent limited studies on the role of cholangioscopy-directed RFA and PDT are encouraging. More extensive studies, including head-to-head trials, are needed to understand the role and possible superiority of POC-directed RFA and PDT in managing unresectable cholangiocarcinoma.

#### 2.1.7 Selective Biliary Canulation

As discussed in prior sections, managing biliary strictures, whether benign or malignant, can often be challenging. A frequent cause of endoscopic failure is the inability to pass a guidewire through the biliary stricture to allow subsequent balloon dilation therapy and endoprosthesis implantation (60). Direct visualization using cholangioscopy has been shown to improve outcomes in such cases. Bokemeyer et al. evaluated the utility of POC-assisted guidewire placement for biliary strictures in 23 patients with previous failed conventional guidewire placement. These 23 patients cumulatively underwent 30 single-operator cholangioscopy procedures with successful guidewire placement in 21 of the 30 procedures (70%) (60). This study also found that digital SOC-assisted guidewire placements were significantly more successful in patients with benign strictures than those with malignant strictures (88.2% vs. 46.2%). Peroral cholangioscopy can thus be an alternative to other, sometimes more invasive treatment options such as percutaneous transhepatic or EUS-guided biliary drainage, that are employed in these circumstances. However, data on using POC for selective biliary canulation is still limited to case series. The incremental clinical gain in many cases should be balanced against the potential for adverse events and additional cost concerns.

### 2.2 Applications of pancreatoscopy

#### 2.2.1 Pancreatoscopy-guided lithotripsy

Traditional ERCP techniques, such as extraction balloons and stone extraction baskets, can have limited success rates of around 50% for pancreatic duct stones, even in expert hands (61). Extracorporeal shock wave lithotripsy (ESWL) is considered the first-line therapy for an obstructed main pancreatic duct (62). It is a very effective method to clear large radio-opaque stones (> 5 mm) in the main pancreatic duct, with a reported success rate ranging from 59% to 76% (63, 64). The availability, high cost, need for multiple sessions and concomitant ERCP, and limited applicability with radiolucent stones are some of the limitations of ESWL (61). Peroral pancreatoscopy-guided (POPS-guided) lithotripsy was first described by Howell et al. in 1999 (65). Electrohydraulic lithotripsy (EHL) or laser lithotripsy (LL) can be used to attain intraductal stone fragmentation. In a systematic review and meta-analysis of 15 studies that evaluated the technical and clinical success rate of POPS-guided lithotripsy for pancreatolithiasis, Guzmán-Calderón et al. reported pooled technical and clinical success rates of 88.1% and 87.1%, respectively (66). This study included 218 patients treated with EHL and 155 patients treated with LL. For POPS-directed EHL, the pooled technical success rate was 90.90% and the pooled clinical success rate was 89.80%, whereas for POPS-directed LL, the pooled technical and clinical success rates were 88.40% and 85.80%, respectively (66). Reported rates of adverse events with POPS-directed lithotripsy for pancreatolithiasis have ranged from 9% to 20% and are comparable to those of ESWL (61, 66, 67). A recent multicenter trial involving 40 patients with chronic pancreatitis evaluated the role of single-operator pancreatoscopy in treating symptomatic pancreatic duct stones. Complete stone clearance was achieved in 90% (36/40) of patients, with a significant improvement in pain after a mean of 1.36 interventions (68). In this study, complete pain relief was reported by 62% of the patients at the 6-month follow-up. The overall rate of adverse events in this study was 12.5%, all of which were managed conservatively (68). Cholangioscopy-directed lithotripsy for large symptomatic pancreatic duct stones could be a promising addition to the current endoscopic armamentarium for managing complex pancreatolithiasis.

#### 2.2.2 Intraductal papillary mucinous neoplasm

**Figure 14:**
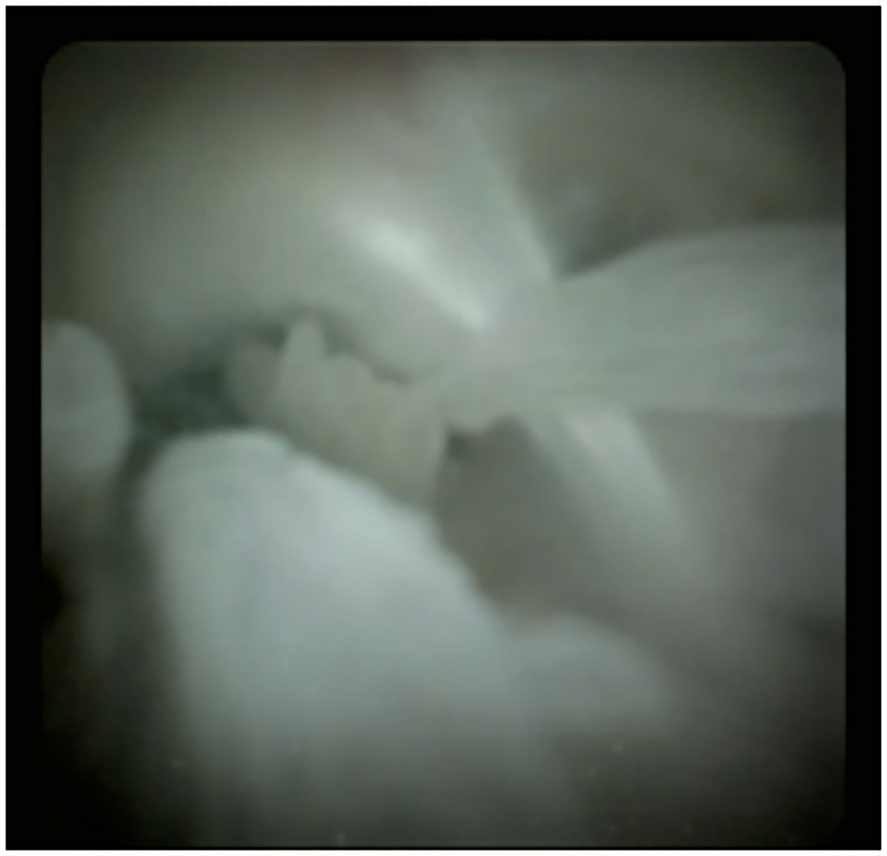
Main-duct IPMN as seen on a pancreatoscopy.

Intraductal papillary mucinous neoplasms (IPMNs) are macroscopic precursor lesions for pancreatic cancer with a variable risk of malignant transformation. They account for 20%–30% of pancreatic cancers (69). IPMNs are characterized by papillary projections of mucin-producing neoplastic epithelium, which causes cystic dilation of the PD (70). They are classified into three main types: main-duct type (MD-IPMN), branch-duct type (BD-IPMN), and mixed-type IMPNs. MD-IPMNs more frequently progress to pancreatic cancer (in 60%–70% of cases) (71–73) than BD-IPMNs or mixed type IMPNs. Most IPMNs detected incidentally on cross-sectional imaging are asymptomatic at diagnosis. Their high incidence and limitations in predicting their natural course make surveillance of IPMNs controversial (69). Diagnostic modalities employed in the evaluation and surveillance of IPMNs include MRI, EUS with EUS-FNA, and ERCP. Imaging findings associated with an increased risk for malignancy are a cyst size ≥3 cm, a dilated main pancreatic duct, and the presence of a solid component (74). The primary role of POPS in IPMN is to confirm the diagnosis in equivocal cases. Hara et al. classified protruding lesions on pancreatoscopy into five groups to discriminate malignant from non-malignant IPMNs, with a reported accuracy of 88% for MD-IPMNs and 67% for BD-IPMNs (75). Intraoperative pancreatoscopy has been shown to change surgical outcomes in patients with mucin-producing tumors of the pancreas (76, 77). POPS has been evaluated to replicate these findings and better define the extent of malignancy to aid in surgical planning. Tyberg et al. demonstrated the utility of pre-surgical “mapping” with digital cholangiopancreatoscopy. In this study, out of a total of 13 patients who underwent POPS, the findings from pancreatoscopy modified the surgical plan with 31% of patients undergoing more extensive surgery, and 31% undergoing less extensive surgery. The overall correlation between endoscopy and surgical histology was 88% (51). In a multicenter Scandinavian study by Vehviläinen et al. on the role of POPS in the preoperative evaluation of suspected MD-IPMNs, clinical management was altered by the findings from pancreatoscopy in 85% of patients (78). In this study, a condition other than IPMN was found that explained main-duct dilation in 28/101 (28%) cases. There was a high incidence of post-POPS pancreatitis reported from this study, with a rate of 20%, including one fatality. The authors noted a decrease in the odds of post-POPS pancreatitis with an increase in main-duct diameter (78). Cholangioscopy is not a standard part of the diagnostic algorithm for the evaluation and management of IPMNs in the currently available guidelines (69). However, with the improvement in technology and accumulating experience, it is anticipated to play an essential role in the future. **Figure 14** shows the appearance of an MD-IPMN on pancreatoscopy.

#### 2.2.3 Pancreatic duct strictures

Evaluating and managing pancreatic duct strictures is typically more challenging than evaluating and managing biliary duct strictures. Given the complexities involved, advanced management of pancreatic duct strictures is restricted to experts in pancreatic endotherapy (63). Common etiologies for pancreatic duct strictures include benign conditions such as chronic pancreatitis, recurrent acute pancreatitis, trauma, surgical complications and pseudocysts; however, they can also be a manifestation of malignancy (79). The treatment of pancreatic duct strictures depends on the etiology, and, if asymptomatic, most benign strictures can be left alone once malignancy is excluded. The conventional endoscopic therapy for symptomatic pancreatic duct strictures comprises pancreatic sphincterotomy followed by the dilation of the pancreatic stricture and placement of pancreatic duct stents (79). Compared to other diagnostic methods, POPS offers the advantage of direct visual inspection with directed biopsies. Findings from pancreatoscopy that suggest pancreatic cancer include erythema, friability, erosions, infiltrative strictures (with near occlusion of the lumen), irregular margins, and signs of external compression with normal mucosa (61). In a study by El Hajj et al. evaluating the role of POPS in suspected pancreatic duct neoplasia, visual impression had a sensitivity, specificity, positive predictive value, negative predictive value, and accuracy of 87%, 86%, 83%, 91%, and 87%, respectively. When combined with POP-guided tissue sampling, these values increased to 91%, 95%, 94%, 93%, and 94%, respectively (80). In a study evaluating indeterminate pancreatic duct strictures in 18 patients, Jung et al. used POPS with cytology and brushings. They confirmed neoplasia in seven patients and chronic pancreatitis in eight. This study reported white-gray smooth narrowing without superficial vessels as a feature characteristic of chronic pancreatitis (81). Image-enhancing technologies such as NBI and confocal laser endomicroscopy (pCLE) can further improve the diagnostic accuracy of POPS in evaluating pancreatic strictures, as demonstrated in the studies by Miura et al. (82) and Itoi et al. (83).

## 3 Future applications

**Figure 15:**
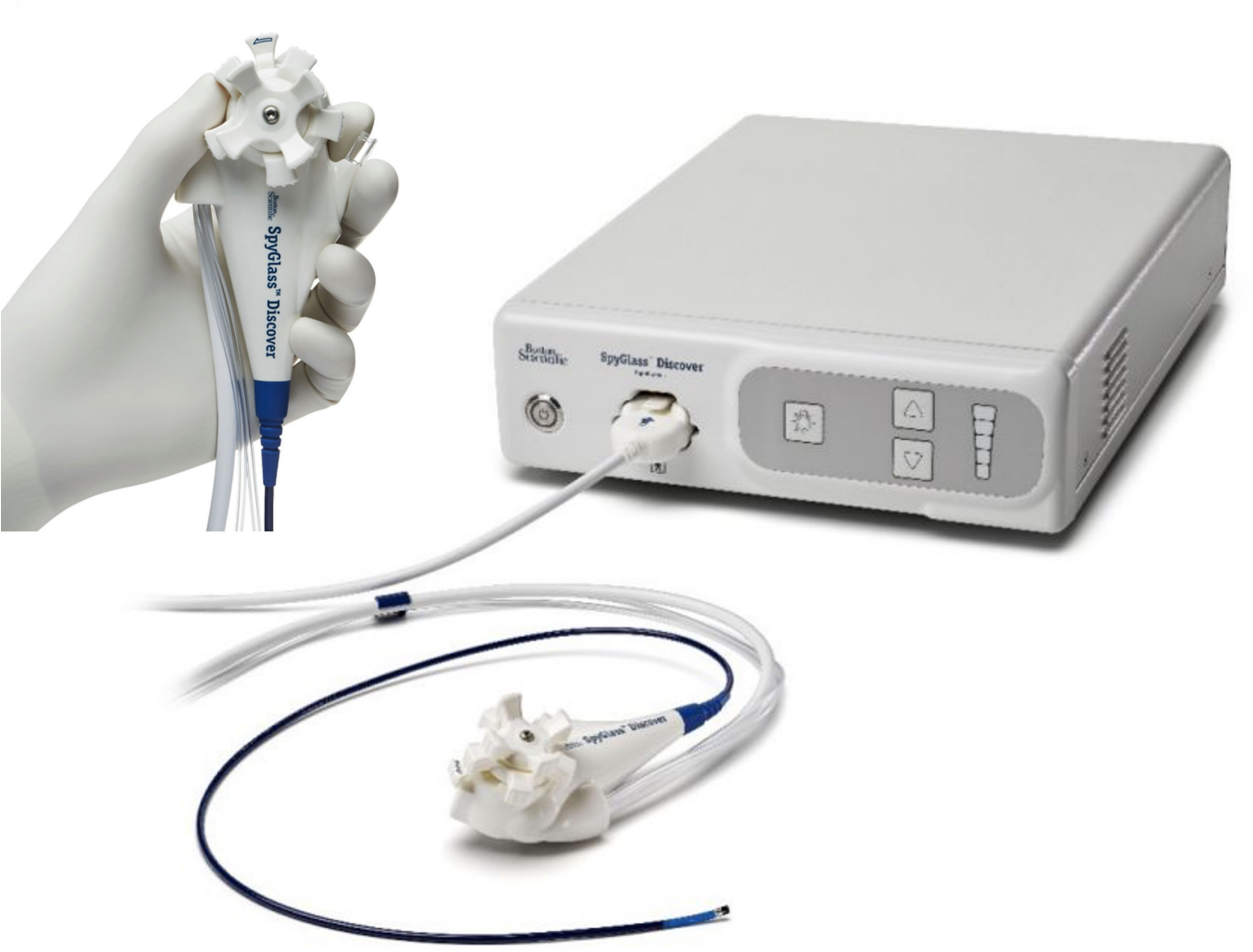
The SpyGlass Discover system.

In addition to the established roles described above, the role of peroral cholangioscopy continues to expand. Multiple case reports have demonstrated its use in retrieving proximally migrated and embedded biliary and pancreatic stents (84–88). The evaluation of haemobilia is another well-documented use of cholangioscopy (89–91). Various other innovative uses of cholangioscopy have been reported in the literature. Endoscopic transpapillary gallbladder drainage (ETGBD) is a well-established alternative therapeutic option for acute cholecystitis in patients who are otherwise not candidates for cholecystectomy. However, it is often challenging to accomplish and requires advanced endoscopic techniques. Yoshida et al. evaluated peroral cholangioscopy-assisted ETGBD and found that it was successful in 11 of 13 patients (84.6%) who had failed conventional ETGBD, thus demonstrating yet another application of this technique (92). ERCP during pregnancy, especially in the first trimester, carries the risk of teratogenicity to the fetus. POCP has been proposed as a radiation-free approach to managing choledocholithiasis in pregnant women while mitigating the radiation risk to the fetus (93). Post-orthotopic liver transplant biliary anastomotic strictures can sometimes be challenging to manage effectively. POC-directed targeted corticosteroid injection as a successful treatment option for refractory anastomotic strictures has been described (94). A novel classification system based on POC findings called the Edmonton classification has been proposed for dominant strictures in PSC (95). Finally, novel therapeutic devices that are small enough to pass through a cholangioscope will create new opportunities for treating ductal pancreaticobiliary diseases. With the development of dedicated devices for various cholangioscopic approaches, such as the SpyGlass™ Discover (**Figure 15**), rendezvous procedures that combine endoscopic cholangioscopy with other approaches could be exciting future options for managing complex biliary pathologies.

Another exciting development in endoscopy is the possibility of using artificial intelligence (AI) technology applied to computer vision to augment our diagnostic capabilities. The AI-aided interpretation of cholangioscopic images in real time has been evaluated to diagnose conditions such as indeterminate or malignant biliary strictures and cholangiocarcinoma (96). Cholangioscopic features such as tumor vessels and papillary projections have been shown to predict a high likelihood of malignancy. AI algorithms that can automatically detect these features have been developed. In a study of 3,920 images from 85 patients, an AI algorithm using a convolutional neural network (CNN) showed a sensitivity of 99.7% and specificity of 97.1% for detecting papillary projections (97). Similarly, another CNN-based AI system reported a sensitivity, specificity, positive predictive value, and negative predictive value of 99.3%, 99.4%, 99.6%, and 98.7%, respectively, for diagnosing tumor vessels in a study of 6,475 images from 85 patients (98). With the further refinement and adoption of AI, which depends on luminal images obtained at the time of cholangioscopy, the role of POCP in aiding the AI-assisted diagnosis of complex biliopancreatic pathologies is expected to increase.

## 4 Limitations

Cholangiopancreatoscopy, particularly POCP, has several advantages and an exciting future, as discussed in this review. This is still a relatively nascent technology that has shown excellent results in experienced hands. However, this complex and technically challenging procedure requires advanced endoscopic skills, and most of the currently available data are from procedures performed in a few high-volume tertiary centers by experienced advanced endoscopists. DPOCP can be challenging, given the need to directly maneuver a flexible slender endoscope into the duodenum and then gain access across the ampulla. This often creates loops that require special maneuvers to reduce and often requires intubating the papilla in a retroflexed position, which can be challenging even for experienced endoscopists. A cumulative sum analysis revealed an initial learning process for endoscopists of nine procedures with significant variation in results, followed by a steady improvement. However, in this study, all the procedures were performed by an experienced endoscopist, and the authors caution that this procedure should be performed only by endoscopists with experience (99). It is also essential to recognize that the overall rates of adverse events are higher with cholangiopancreatoscopy than with ERCP alone (7% vs. 2.9%) (2, 100). Air embolization, bile duct perforation, pancreatitis, cholangitis, and bleeding are the most common complications associated with a cholangioscopy or pancreatoscopy (100). The risk for air embolization persists even when mitigating measures, such as CO_2_ insufflation or saline irrigation, are followed. When pancreatoscopy is performed with concurrent intraductal ultrasound, the rate of pancreatitis has been reported to be as high as 7% (75). Although cholangioscopy is an excellent tool for diagnosing and navigating complex biliary strictures or for selective bile duct canulation, it is essential to realize that there are limitations to this technique. POC can fail to diagnose strictures caused by extrinsic compression which can be from benign or malignant etiology. For example, one study found that sensitivity of POC was 84% for diagnosing intrinsic malignancies and 62% for diagnosing extrinsic strictures (101). POC using currently available devices can also be limited in evaluating small-caliber proximal intrahepatic bile ducts and narrow strictures that might prove impossible to traverse.

Yet another major concern with the newer single-use peroral cholangioscopes is cost-effectiveness and environmental impact. Cost-effectiveness is multifactorial and its calculation includes the cost of endoscopes and processors, which is to be balanced with the cost of reprocessing, maintenance, and the repair of reusable scopes, including the cost of human labor, with most data coming from studies on disposable single-use duodenoscopes (102). The high rates of exogenous transmission of multidrug-resistant organisms from the use of contaminated reusable endoscopes, particularly duodenoscopes, is an emerging concern (103). Recently the US FDA has recommended that hospitals and endoscopy facilities complete the transition to innovative duodenoscope designs due to concerns about the inadequacy of reprocessing in preventing cross-contamination (104). Single-use cholangioscopes are part of this disposable endoscope technology, but their environmental impact and cost-effectiveness continue to be an active area of ongoing research.

## 5 Conclusions

Cholangiopancreatoscopy is an effective diagnostic and therapeutic method to evaluate and treat biliopancreatic pathologies, with an acceptable safety profile. Peroral cholangiopancreatoscopy has made rapid progress in the past decade. We have provided a comprehensive review of the advances in digital endoscopic imaging technologies and the role of cholangiopancreatoscopy in managing complex biliary and pancreatic disorders. POC still has its limitations, but in this era of rapid technological advancements, as the cholangioscope is developed and further clinical experience is gained, we anticipate the continued expansion of its applications.

## Author contributions

The authors of this study confirm the contributions to the article were as follows: HG, MD—manuscript design, literature review, writing, and submission; NS, MD—topic conception, expert review, images, and editing. All authors contributed to the article and approved the submitted version.
